# The prognostic value of Tiam1 protein expression in head and neck squamous cell carcinoma: a retrospective study

**DOI:** 10.1186/s40880-015-0053-3

**Published:** 2015-09-14

**Authors:** Hang Yang, Yu-Chen Cai, Ye Cao, Ming Song, Xin An, Yi Xia, Jing Wei, Wen-Qi Jiang, Yan-Xia Shi

**Affiliations:** State Key Laboratory of Oncology in South China, Collaborative Innovation Center for Cancer Medicine, Sun Yat-sen University Cancer Center, Guangzhou, Guangdong 510060 P. R. China; Department of Medical Oncology, Sun Yat-sen University Cancer Center, Guangzhou, Guangdong 510060 P. R. China; Clinical Trial Center, Sun Yat-sen University Cancer Center, Guangzhou, Guangdong 510060 P. R. China; Department of Head and Neck Surgery, Sun Yat-sen University Cancer Center, Guangzhou, Guangdong 510060 P. R. China; Department of Medical Oncology, Ruikang Hospital, Guangxi Traditional Chinese Medical University, Nanning, Guangxi 530000 P. R. China

**Keywords:** Tiam1, Head and neck squamous cell carcinoma, Prognosis

## Abstract

**Introduction:**

Head and neck squamous cell carcinoma (HNSCC) is a common cancer worldwide and has a poor prognosis. A biomarker predicting the clinical outcome of HNSCC patients could be useful in guiding treatment planning. Overexpression of the T lymphoma invasion and metastasis 1 (Tiam1) protein has been implicated in the migration and invasion of neoplasms. However, its role in HNSCC progression needs to be further validated. We detected the expression of Tiam1 in normal and tumor tissues and determined its association with clinical outcomes in patients with HNSCC.

**Methods:**

We measured the expression of Tiam1 in normal and cancerous tissue samples from the patients with HNSCC treated at Sun Yat-sen University Cancer Center between 2001 and 2008. The Tiam1 expression was scored from 0 to 12 based on the percentage of positively stained cells and the staining intensity. We then determined the diagnostic performance of this score in predicting overall survival (OS) and disease-free survival (DFS).

**Results:**

Of the 194 evaluable patients, those with advanced disease, lymph node metastasis at diagnosis, and recurrence or metastasis during follow-up had a higher tendency of having high Tiam1 expression as compared with their counterparts (*P* < 0.05). The proportion of samples with high Tiam1 expression was also higher in cancerous tissues than in non-cancerous tissues (57.7% vs. 13.9%, *P* < 0.001). Cox proportional hazards regression analysis revealed that Tiam1 expression scores of 5 and greater independently predicted short OS and DFS.

**Conclusion:**

The Tiam1 expression is shown as a promising biomarker of clinical outcomes in patients with HNSCC and should be evaluated in prospective trials.

## Background

Head and neck squamous cell carcinoma (HNSCC) is the sixth most common cancer worldwide, with approximately 600,000 new cases diagnosed per year [1–3]. Despite remarkable improvements in diagnosis and treatment, a high recurrence rate has kept the 5-year survival rate at approximately 50% for many years [1, 4]. A biomarker predicting the clinical outcome of HNSCC patients could be useful in guiding treatment.

The Rho-like proteins (Rho, Rac, and Cdc42) are essential regulators of cytoskeleton dynamics [5] and are crucial for malignant cell progression such as mitogenesis, kinase cascade activation, transcriptional activation, and DNA synthesis stimulation [6–8]. The expression of certain Rho subfamily proteins is elevated in HNSCC cell lines, and RhoA has long been thought to be a promising biomarker in HNSCC [9]. Furthermore, the overexpression of RhoC was greater in advanced HNSCC than in early disease [10]. The activation of Cdc42 is also critical for the invasion of HNSCC cells and is mediated by galectin-1 (Gal-1) and CCL19-induced chemokine receptor 7 (CCR7) [11]. Additionally, Gal-1 overexpression enhances Cdc42 activation and increases lung metastasis in nude mice [12]. Enhanced Rac1 activation in HNSCC cell lines is also associated with a highly invasive and motile tumor cell phenotype [13].

Cycling between the inactive [guanosine diphosphate (GDP)-bound] and active [guanosine triphosphate (GTP)-bound] states of Rho subfamily proteins is regulated by the guanine nucleotide exchange factors (GNEFs) [14]. T lymphoma invasion and metastasis 1 (Tiam1), a member of Dbl gene subfamily of the GNEFs family, is first identified in T lymphocytes [15]. It regulates general Rho proteins [16] with multiple cellular effects [17, 18]. Tiam1 is also important in regulating cell growth, differentiation, and motility [19]. Patel et al. [13] reported that most HNSCC cells exhibited obviously high levels of Rac1 in the active state, and Supriatno et al. [20] elaborated that Tiam1 depletion reduced the migration of oral cancer cells. Moreover, Tiam1 expression affects the migration and invasion of many neoplasms, and high Tiam1 expression has indicated a poor prognosis in various tumors such as nasopharyngeal carcinoma [21, 22], primary gallbladder carcinoma [23], hepatocellular carcinoma [24, 25], papillary thyroid carcinoma [26], esophageal squamous cell carcinoma [27], retinoblastoma [28], and prostate carcinomas [29].

We found only one study that specifically examined Tiam1 expression in laryngeal and hypopharyngeal carcinomas. In tissue samples from 119 patients with HNSCC, Wang et al. [30] found that high Tiam1 expression was significantly associated with lymph node metastasis, clinical disease stage, histological tumor grade, recurrence, and short overall survival (OS) and disease-free survival (DFS). In a larger study with longer follow-up, we sought to confirm whether the expression of Tiam1 in several types of HNSCC, especially oral cancer, was associated with disease progression and long-term outcomes.

## Methods

### Patients and specimens

HNSCC patients treated at the Department of Head and Neck Surgery of Sun Yat-sen University Cancer Center (Guangzhou, China) between January 2001 and December 2008 were selected retrospectively and randomly by stratified sampling. We defined HNSCC as cancer of the oral cavity, glottis, and supraglottic larynx. The selection criteria were as follows: (1) patients had undergone complete resection, with or without unilateral or bilateral neck dissection; (2) the diagnosis of HNSCC was confirmed by pathology; (3) complete follow-up data and pathologic specimens were available. Patients were ineligible if they had metastasis at the time of diagnosis (stage IVc), had undergone radiotherapy and/or chemotherapy before surgery, or had other concomitant malignant neoplasms or organ disorders. The records of the patients selected for analysis were reviewed, and cancers were restaged according to the 2010 American Joint Committee on Cancer TNM Staging Manual. All hematoxylin and eosin-stained slides were reviewed to verify the diagnosis and adequacy of the specimen for analysis. The appropriate paraffin-embedded specimen blocks for each patient were obtained from the Pathology Department. When possible, adjacent non-cancerous tissue specimens were also processed and compared with cancerous specimens as matched pairs.

### Staining and evaluation

The paraffin-embedded blocks were dewaxed and rehydrated, and endogenous peroxidase activity was blocked with 0.3% H_2_O_2_ in methanol. The slides were boiled in ethylene diaminetetraacetic acid (EDTA) on high power for 5 min and medium–low for 20 min in a microwave for antigen retrieval. Non-specific binding was inhibited with normal goat serum. Then, a primary Tiam1 antibody (sc-872, Santa Cruz Biotechnology, Santa Cruz, CA, USA) was used at 1:125 and stored overnight at 4°C. Subsequently, the slides were incubated with a goat anti-rabbit secondary antibody at 37°C for 30 min. Horseradish peroxidase was applied. Finally, hematoxylin was used to counterstain the nuclei. The negative control was created by omitting the primary antibody.

All slides were interpreted by two independent pathologists in a double-blinded manner. The percentage of cells with positive staining was scored as follows: a positive rate of 0% was scored 0, 1%–10% scored 1, 11%–50% scored 2, 51%–80% scored 3, and >80% scored 4. The intensity was scored 0 for no staining, 1 for weak staining (light yellow), 2 for moderate staining (yellowish brown), and 3 for strong staining (brown) [29]. The scores of proportion and intensity of positively stained tumor cells were multiplied to evaluate the final Tiam1 expression score, which ranged from 0 to 12. If the discrepancy between the two scores for the same specimen was 6 or larger, the pathologists re-evaluated the slide and reached a consensus on the score.

### Patient follow-up

All patients had follow-up data through April 2014. Metastasis and recurrence were diagnosed based on clinical examination, imaging assessment, or operation and pathologic examination. OS was calculated from the time of diagnosis to the date of death or the last follow-up visit. DFS was defined as the time between diagnosis and relapse.

### Statistical analysis

All data were analyzed with the SPSS 19.0 statistical software package (SPSS, Inc., Chicago, IL, USA). The receiver operating characteristic (ROC) curve and Youden Index (YI = sensitivity + specificity − 1) [31] were computed to determine the optimal cut-off point for distinguishing between high and low Tiam1 expression. The Chi Square test was used to assess the associations between Tiam1 expression and other characteristics of patients. Survival was analyzed with univariate analysis and Kaplan–Meier survival curves. Univariate associations with *P* values less than 0.05 were considered in multivariate analysis and Cox proportional hazards models. A two-sided *P* value less than 0.05 was considered significant.

## Results

### Patient characteristics

The 194 eligible patients with stages I-IVb HNSCC had a median age of 54 years (range 25–86 years); 150 were men (Table 1). Of the 194 patients, 156 had oral cancers, 11 had supraglottic cancers, and 21 had glottic cancers. Adjuvant radiotherapy was given to 34 (17.5%) patients. The median follow-up time was 79 months (range 3–168 months). Only 14 (7.2%) patients were lost to follow-up. As of the last follow-up visit, 116 (59.8%) patients had local or distant relapse events. The disease-specific mortality was 41.8% (81/194).

**Table 1:** Associations between T lymphoma invasion and metastasis 1 (Tiam1) expression and clinicopathologic characteristics of the 194 patients with head and neck squamous cell carcinoma (HNSCC)

Characteristic	Tiam1 expression (cases)^a^		*P* value
	Low	High	
Total	82	112	
Age (years)			
≤50	29	46	0.836
51–60	27	34	
61–70	19	25	
>70	7	7	
Gender			
Male	59	91	0.127
Female	23	21	
Tobacco use			
No	38	38	0.080
Yes	44	74	
Alcohol use			
No	62	85	0.964
Yes	20	27	
Histological differentiation			
Well	48	67	0.289
Moderate	28	30	
Poor	6	15	
Chronic illness			
No	66	90	0.982
Yes	16	22	
T stage			
T1/T2	73	90	0.104
T3/T4	9	22	
Lymph node metastasis			
No	65	66	0.003
Yes	17	46	
Clinical stage			
I/II	62	57	<0.001
III/IV	20	55	
Tumor position			
Oral cavity	71	85	0.064
Buccal mucosa	7	6	
Floor of mouth	3	8	
Anterior tongue	51	56	
Alveolar ridge	10	15	
Glottic larynx	8	19	
Supraglottic larynx	3	8	
Disease recurrence			
No	45	33	<0.001
Yes	37	79	

^a^Tiam1 expression is scored from 0 to 12. Scores from 0 to 4 indicate low expression; scores of 5 or greater indicate high expression. See the text for details

### Cut-off score selection

**Fig. 1:**
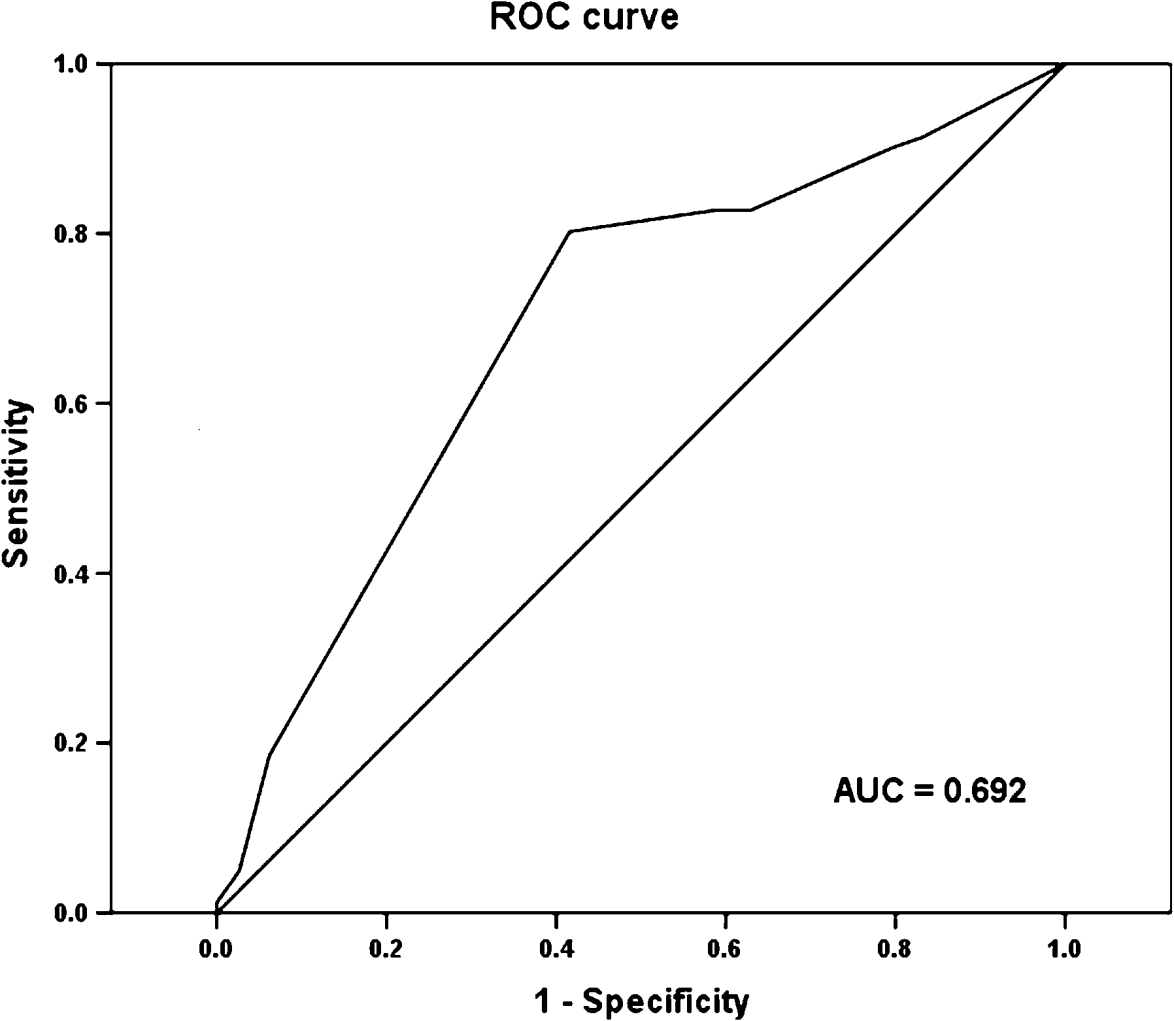
Selection of the cut-off score for T lymphoma invasion and metastasis 1 (Tiam1) expression by the receiver operating characteristic (ROC) analysis. The area under the curve (AUC) is 0.692. An expression score of 5 maximizes the Youden Index [sensitivity (0.802) + specificity (0.584) − 1 = 0.386]

The ROC curve for the scores of Tiam1 expression was plotted to select the appropriate cut-off score (Fig. 1). The area under the curve was 0.692 [*P* < 0.001, 95% confidence interval (CI) 0.615–0.768]. A Tiam1 score of 5 maximized the Youden Index [sensitivity (0.802) + specificity (0.584) − 1 = 0.386] as the optimal cut-off score. Thus, cases were divided into low (score < 5) and high (score ≥ 5) expression groups.

### Tiam1 expression and clinicopathologic factors

**Fig. 2:**
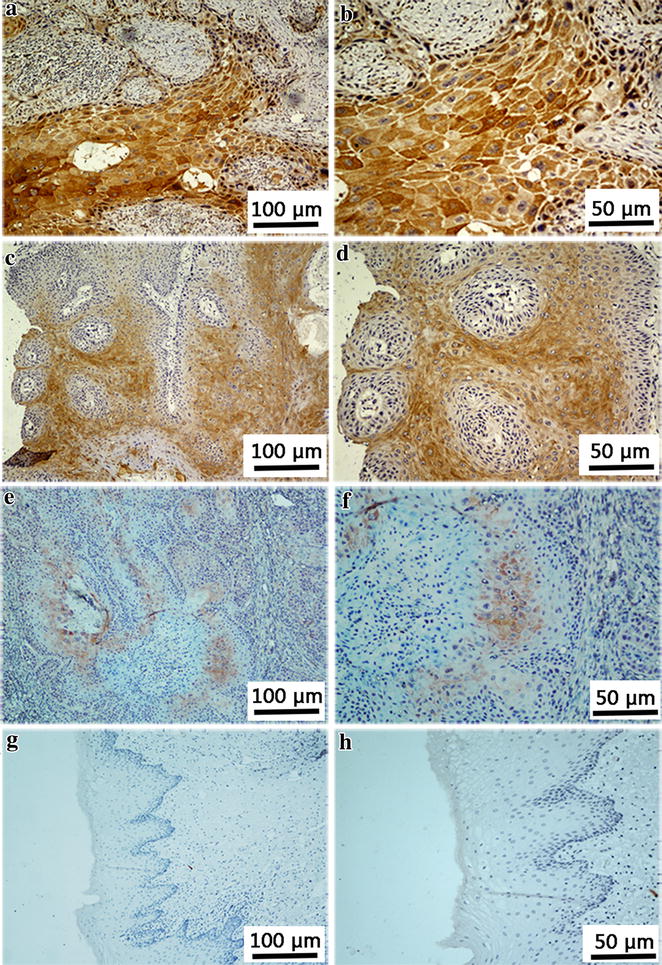
Tiam1 expression in tumor samples and non-cancerous tissue samples. Tiam1 protein staining mainly distributes in the cytoplasm. **a** (100×) and **b** (200×), strong staining of Tiam1 protein in tumor cells. **c** (100×) and **d** (200×), moderate staining in tumor cells. **e** (100×) and **f** (200×), weak staining in tumor cells. **g** (100×) and **h** (200×), no Tiam1 protein staining is present in the non-carcinoma epithelium

The Tiam1 protein was located in the cytoplasm (Fig. 2). The high expression rate was higher in tumor tissues than in the matched non-cancerous tissues (57.7% vs. 13.9%, *P* < 0.001). High Tiam1 expression was significantly associated with relapse (*P* < 0.001), lymph node metastasis (*P* = 0.003), and stage III/IV cancers (*P* < 0.001) (Table 1). The association between Tiam1 expression and tumor position was marginally significant (*P* = 0.064).

### Predictive value of the Tiam1 expression score

**Fig. 3:**
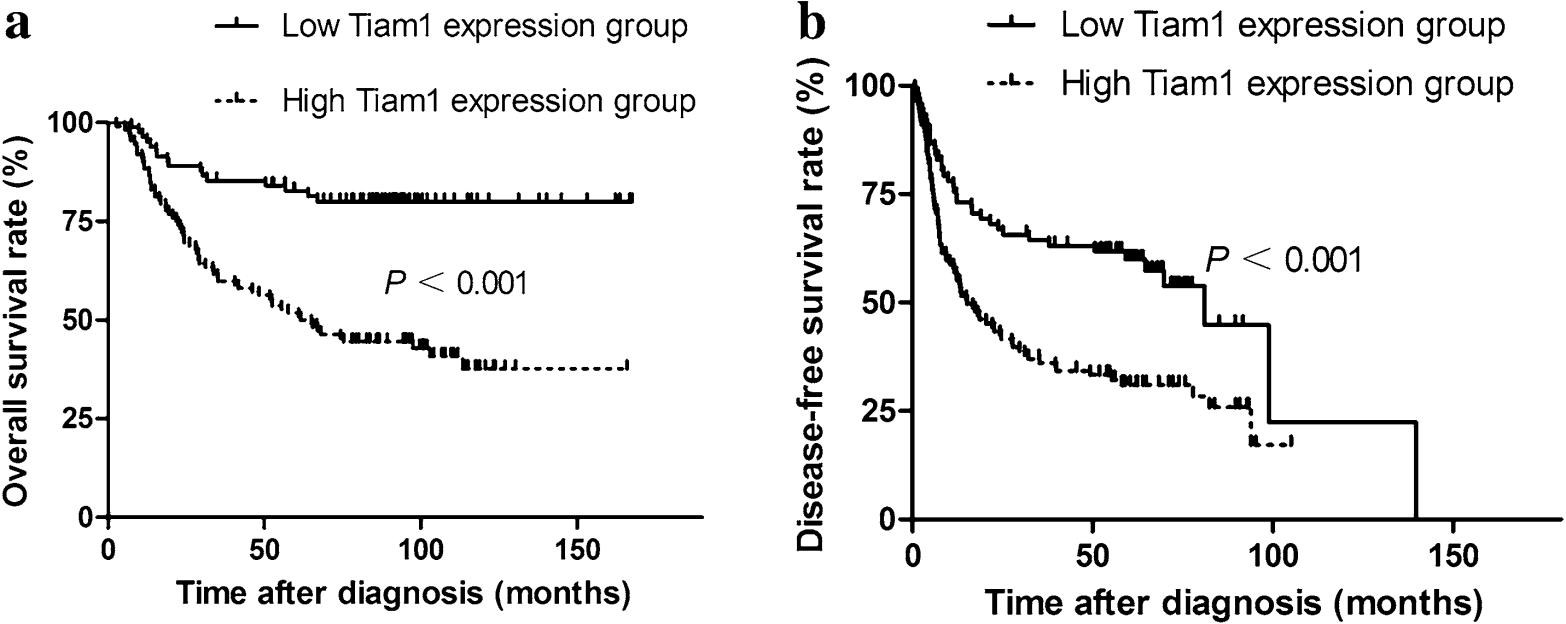
Survival curves of 194 patients with head and neck squamous cell carcinoma, stratified by Tiam1 expression score. **a** overall survival; **b** disease-free survival

The 1-, 3-, and 5-year OS rates of all patients were 91.8, 70.5, and 64.7%, respectively. The 1-, 3-, and 5-year DFS rates were 64.8%, 48.1%, 43.2%, respectively. The relapse rate was higher in the high Tiam1 expression group than in the low Tiam1 expression group (70.5% vs. 45.1%, *P* < 0.001), as was the mortality (58.0% vs. 19.5%, *P* < 0.001). In the high Tiam1 expression group, the median OS was 61.5 months (95% CI 41.3–81.7 months), and the median DFS was 15.2 months (95% CI 6.7–23.7 months). The 5-year OS and DFS rates were 51.8% and 31.0%, respectively. The low Tiam1 expression group had a rather improved long-term survival: the 5-year OS and DFS rates were 82.7% and 60.0%, respectively. High Tiam1 expression predicted short OS and DFS (both *P* < 0.001, Fig. 3). In univariate survival analysis, patients using tobacco and alcohol with poorly differentiated tumors, lymph node metastasis, and stage III or IV disease had shorter OS survival, and patients with poorly differentiated tumors, no neck dissection, advanced disease, and oral cavity cancer had shorter DFS than their counterparts (Table 2). Cox proportional hazards analysis revealed that high Tiam1 expression independently predicted short OS and DFS (*P* < 0.001), as did advanced disease and alcohol use (Table 3).

**Table 2:** Univariate survival analysis of 194 HNSCC patients

Variable	OS		DFS	
	RR (95% CI)	*P* value	RR (95% CI)	*P* value
Male	1.49 (0.84–2.65)	0.173	1.28 (0.81–2.04)	0.295
Chronic illness	1.23 (0.72–2.10)	0.445	0.95 (0.59–1.52)	0.819
Tobacco use	1.59 (0.99–2.55)	0.051	1.44 (0.97–2.14)	0.067
Alcohol use	1.95 (1.23–3.09)	0.004	1.46 (0.98–2.18)	0.065
Age >70 years	1.18 (0.51–2.71)	0.698	1.00 (0.49–2.05)	0.991
Age <45 years	1.08 (0.65–1.81)	0.771	1.04 (0.67–1.59)	0.876
Poor tumor differentiation	2.02 (1.30–3.13)	0.001	1.96 (1.35–2.83)	<0.001
Without neck dissection	0.79 (0.47–1.31)	0.357	1.54 (1.04–2.27)	0.029
Tiam1 overexpression	3.67 (2.12–6.34)	<0.001	2.05 (1.38–3.05)	<0.001
Stage III/IV disease	3.39 (2.16–5.31)	<0.001	2.36 (1.63–3.42)	<0.001
Oral cavity cancer	1.13 (0.68–1.90)	0.630	2.48 (1.68–3.67)	<0.001
Adjuvant radiotherapy	0.64 (0.38–1.08)	0.091	0.86 (0.54–1.37)	0.515

*OS* overall survival, *DFS* disease-free survival, *RR* relative risk, *CI* confidence interval

**Table 3:** Multivariate survival analysis of 194 HNSCC patients

Variable	OS		
	RR	95% CI	*P* value
Tobacco use	1.04	0.63–1.74	0.870
Alcohol use	1.71	1.05–2.80	0.032
Poor tumor differentiation	1.54	0.97–2.45	0.067
Tiam1 overexpression	3.00	1.71–5.29	<0.001
Stage III/IV disease	2.16	1.32–3.51	0.002

Variable	DFS		
	RR	95% CI	*P* value
Poor tumor differentiation	1.412	0.932–2.140	0.103
Without neck dissection	1.703	1.083–2.678	0.021
Tiam1 overexpression	1.709	1.129–2.586	0.011
Stage III/IV disease	2.008	1.299–3.105	0.002
Oral cavity cancer	1.437	0.900–2.293	0.129

Abbreviations as in Table 2

## Discussion

We confirmed that a larger proportion of HNSCC tissue samples had high Tiam1 expression compared with non-cancerous tissue samples (57.7% vs. 13.9%, *P* < 0.001). High Tiam1 expression was also associated with higher relapse rates, higher mortality, lymph node metastasis, and stage III/IV cancers as compared with low Tiam1 expression (all *P* < 0.05).

The Rho-like proteins are crucial for malignant cell progression [6, 8], and many Rho subfamily proteins have been thought to be biomarkers of HNSCC [9–12]. Tiam1 is a general guanine nucleotide exchange factor that regulates Rho proteins with multiple effects [17, 18]; thus, alterations in its expression might contribute to tumor occurrence, progression, and migration [18, 19, 27].

The prognosis of HNSCC patients is mainly determined by disease stage, lymph node status, and other advanced disease characteristics at diagnosis [32]. Through a great number of epidemiologic investigations, betel nut chewing [33], tobacco use, and alcohol intake [34, 35] have been established as major risk factors for HNSCC. Strong links have also been found between HNSCC and human papillomavirus (HPV) infection [36], Epstein-Barr virus (EBV) infection [37], and epidermal growth factor receptor (EGFR) overexpression [38]. In the present study, both the univariate and multivariate analyses confirmed that alcohol use and stage III/IV cancer are independent risk factors for poor prognosis. Due to the insufficient number of cases and restrictions in experimental conditions, we did not find a relationship between any other characteristics and long-term survival. In our study, Cox regression analysis showed that Tiam1 overexpression significantly predicted short DFS and OS (both *P* < 0.05). Our data confirmed that Tiam1 overexpression indicates a poor prognosis for HNSCC patients. Wang et al. [30] also found that high Tiam1 expression was associated with short DFS and OS in patients with HNSCC. There are still differences between our study and their study. On the one hand, their samples comprised patients with laryngeal and hypopharyngeal carcinoma, whereas we selected mainly patients with oral cancer and a few people with glottal and supraglottic laryngeal cancer. On the other hand, our study had a longer follow-up and larger sample size than theirs.

On the basis of these results, we boldly considered that Tiam1 overexpression in HNSCC patients might be used as a promising biomarker to identify high-risk patients to aid in the design of optimal individual treatments. However, we failed to develop a prospective study. As a retrospective investigation, our study is limited by the deficiency of large-scale screening. Although we found a possible relationship between Tiam1 overexpression and the invasiveness and metastasis of HNSCC, the underlying mechanisms are unclear.

## Conclusions

Our findings confirm that high Tiam1 expression in the carcinomas of the oral cavity, glottis, and supraglottic larynx predicts poor clinical outcomes, suggesting that Tiam1 might be a new molecular biomarker of this disease. If so, patients with high Tiam1 expression should receive more aggressive therapy and closer follow-up. Tiam1 may also represent a new molecular target for tumor therapy. Tiam1 expression shows promise as a biomarker in patients with HNSCC and should be evaluated in prospective trials.
